# Molecular Junctions Enhancing Thermal Transport within Graphene Polymer Nanocomposite: A Molecular Dynamics Study

**DOI:** 10.3390/nano11102480

**Published:** 2021-09-23

**Authors:** Alessandro Di Pierro, Bohayra Mortazavi, Alberto Fina

**Affiliations:** 1 Dipartimento di Scienza Applicata e Tecnologia, Politecnico di Torino, Alessandria Campus, Viale Teresa Michel 5, 15121 Alessandria, Italy; dipierro.alessandro@gmail.com; 2 Department of Mathematics and Physics, Leibniz Universität Hannover, Appelstraße 11, 30167 Hannover, Germany; bohayra.mortazavi@gmail.com

**Keywords:** molecular junctions, thermal boundary resistance, thermal conductivity, graphene, polymer nanocomposites

## Abstract

Thermal conductivity of polymer-based (nano)composites is typically limited by thermal resistances occurring at the interfaces between the polymer matrix and the conductive particles as well as between particles themselves. In this work, the adoption of molecular junctions between thermally conductive graphene foils is addressed, aiming at the reduction of the thermal boundary resistance and eventually lead to an efficient percolation network within the polymer nanocomposite. This system was computationally investigated at the atomistic scale, using classical Molecular Dynamics, applied the first time to the investigation of heat transfer trough molecular junctions within a realistic environment for a polymer nanocomposite. A series of Molecular Dynamics simulations were conducted to investigate the thermal transport efficiency of molecular junctions in polymer tight contact, to quantify the contribution of molecular junctions when graphene and the molecular junctions are surrounded by polydimethylsiloxane (PDMS) molecules. A strong dependence of the thermal conductance was found in PDMS/graphene model, with best performances obtained with short and conformationally rigid molecular junctions. Furthermore, the adoption of the molecular linkers was found to contribute additionally to the thermal transport provided by the surrounding polymer matrix, demonstrating the possibility of exploiting molecular junctions in composite materials.

## 1. Introduction

Thermally conductive polymer-based materials are of interest in several thermal management applications, owing to the unique set of properties of polymeric materials, coupling excellent chemical resistance in harsh environment, mechanical flexibility, and ease of processing into complex shapes [1,2]. As polymers typically have low thermal conductivity, the inclusion of conductive particles is widely used to enhance the heat conduction properties [2,3,4]. Conductive nanoparticles were also exploited to prepare polymer nanocomposites, based on the high thermal conductivity values reported for individual nanoparticles, as well as the possibility of producing percolating networks at low particle loading, given their high aspect ratio. However, thermal conduction in polymer nanocomposites depends on several parameters including the defectiveness and geometrical features of the nanoparticles, the particle loading, their degree of dispersion, orientation, etc. [5,6,7,8]. Furthermore, the formation of a thermally efficient percolating network is strongly limited by the thermal resistances across the interfaces, which becomes dominant when dispersing nanoparticles with high surface area, such as graphene and related materials [3]. Indeed, heat transfer depends on the thermal resistance that rises across the polymer/particles interfaces as well as in the contacts between particles in percolating networks. Despite the enhancement of polymer/graphene interface was proposed to enhance the overall heat transfer in nanocomposites [9,10], the very low mean free path for phonons in the polymer (a few angstroms) [3] compared to the mean free path on graphene (hundreds of nanometers) [11] strongly limits the efficiency of this approach. While the decrease in interfacial resistance between polymer ad conductive particles may contribute to the thermal efficiency in materials with dispersed isolated conductive particles, in the presence of a percolation network of conductive particles, thermally inefficient contacts between particles appear to represent a significant bottleneck in the overall thermal transfer. To overcome this limitation, the adoption of molecular junctions between particles have been evaluated in the literature, both theoretically and experimentally.

Thermal Boundary Conductance (TBC) at particle–particle contacts was numerically studied with different computational tools by various authors, either between carbon nanotubes and graphene platelets, functionalized with small bridging groups [12,13,14,15], short alkyl chains [16,17] or benzene [18]. A TBC increase and a reduction in thermal jump across the junction were generally described. More recently, the effect of chain length and chemical structure of molecular junctions on their thermal conductance was systematically studied, both via Molecular Dynamics (MD) and Density Functional Theory (DFT) [19,20]. Experimental investigation of molecular junctions was also explored. Han et al. [21] first reported aminosilane functionalization of graphene related materials for the application in heat management in electronic and we recently reported edge-functionalized graphene nanoplates for the preparation of thermally conductive nanopapers [22]. While the literature demonstrates enhancements in thermal transport between free-standing particles [23,24], to the best of the authors’ knowledge, no investigation of molecular junctions was previously reported for thermal junctions embedded within a polymer. In fact, in a polymer matrix, the interaction between the surface of graphene flakes and adsorbed macromolecules also allows heat transfer between the conductive particles and the surrounding polymer, although the relatively high interfacial resistance [25]. Even in the presence of molecular junctions connecting two adjacent conductive particles, the presence of polymer chains around the junction between two graphene sheets is expected to contribute to the local thermal transfer within the polymer matrix. Within this scenario, the present paper aims to quantify the contribution of molecular junctions when the graphene and the molecular junctions are surrounded by a model polymer, representing phenomena occurring during heat transfer across polymer nanocomposites.

## 2. Methods

**Figure 1:**
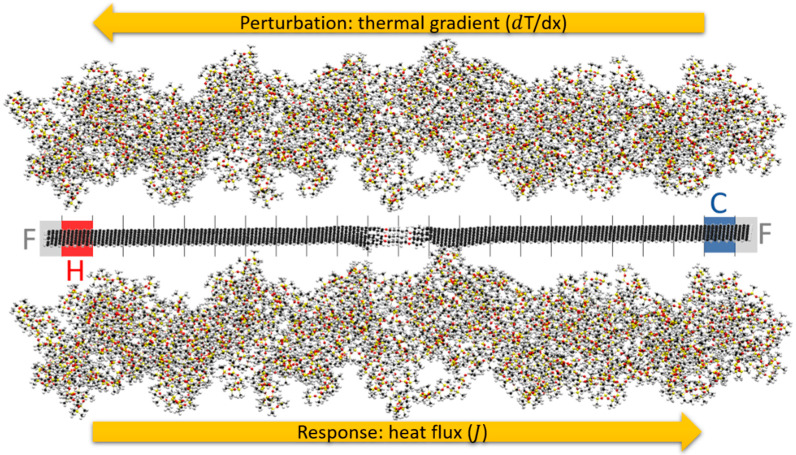
The components of the simulated model. In grey: F as fixed atoms, blue: C as cold reservoir (Nosé–Hoover thermostated region), red: H as hot reservoir (Nosé–Hoover thermostated region), the vertical black lines separate the 22 thermal slabs. Fixed atoms are not counted as thermal slabs.

Classical Molecular Dynamics (MD) has become a popular method to investigate dynamic properties of materials at the atomistic scale [26]. It is based on classical physics, and from early studies on gases from the 1950s [27,28] and nowadays it represent an established technique to study dynamic phenomena occurring at the atomistic scale. The study of condensed matter, such as polymers, also gains advantages from MD simulations because chemical phenomenon occurs at time and size scales that MD is capable to reproduce. An established way to employ MD simulations to study thermal properties of materials is through the Non-Equilibrium MD (NEMD) approach. The idea behind NEMD is to reproduce at the atomistic level the heat transfer conditions that occur at the macroscopic scale, by the application of a perturbation to the system, such as a steady thermal gradient inside the material and measure the response, as internal heat flux. This method, sometimes called the “direct method” in MD simulations, is even suitable in case of inhomogeneities, or interfaces or lattice defects. In this work, we considered different interfaces within the material, and NEMD was found to be a valuable technique to screen thermal properties of different chemical species. In our layout, depicted in Figure 1, the simulation followed a stepwise scheme: at the beginning of the simulation, a 500 ps NPT stage at 300 K and 1.5 GPa was performed bring the system close to the PDMS actual density, allowing the PDMS polymer and the graphene junction to became a dense system. Then, the whole system was relaxed, to reduce the potential energy to a “ground state”, i.e., a local minimum in the potential energy. This initial condition was met through a thermal equilibration period of 250 ps in NVT ensemble at 300 K. It followed a thermostated preheating stage where the perturbation was applied to the graphene. In this stage, the 10 Å long regions constituting the ends of the graphene slab were fixed to avoid motion and at the same time, two Nosé–Hoover thermostats, set at 330 K and 300 K were applied to the thermal slabs Hot and Cold, respectively, adjacent to the fixed atoms’ regions. The rest of the graphene and junction running in NVE were split into other 20 thermal slabs along the x coordinate to calculate the temperature inside the model [29].

After the transitory thermostated pre-heating stage, the system runs flawlessly in steady-state, and for the following 2.5 ns the information of the thermal energy exchanged trough the thermostats and temperature of the slabs [30,31] was computed. The thermal energy flowing through the thermostats was calculated from the slope of the energy versus time plot [29], while the slab temperatures were computed from the time-averaging the instantaneous local kinetic temperature [30,31] by computing Equation (1).

(1) $$T_{i}=\frac{2}{3N_{i}k_{B}}\underset{j}{\sum }\frac{p_{j}^{2}}{2m_{j}}$$

where, *T_i_* is the temperature of *i*th slab portion, *N_i_* is the number of atoms in *I*th group, *k*_B_ is the Boltzmann’s constant, *m_j_* and *p_j_* are atomic mass and momentum of atom *j*, respectively. Within this method, longer simulation time assures higher accuracy of the temperature calculation thanks to the longer sampling time. From the plot of the average temperature of the slabs, as a function of the coordinate displacement, it is possible to evaluate the thermal jump across the junction. As a consequence, the thermal conductance can be computed through Equation (2),

(2) $$G_{c}=\frac{\dot{q}}{A\cdot \Delta T}$$

where *G_c_* is the thermal boundary conductivity, $\dot{q}$ the heat flux flowing into the material, A the area of the interface and Δ*T* the temperature across the interface.

## 3. Modelling

**Figure 2:**
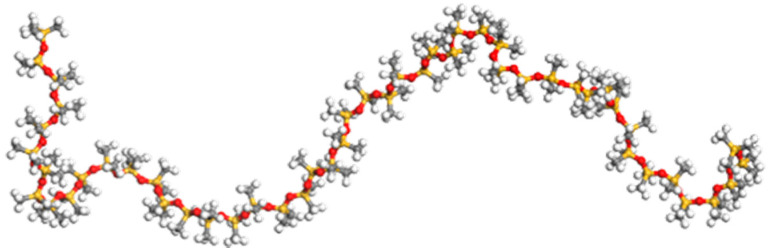
Representation of a PDMS single chain. Color codes: carbon in grey, hydrogen in white, oxygen in red and silicon in yellow.

The largest subset of the atoms constituting the simulation belongs to the Polydimethylsiloxane (PDMS) polymer which surrounds the junction between two graphene foils. The design of the PDMS mass, made up of 2 blocks of 34 molecules (totaling 34,476 atoms), initiated by the creation of a 49-monomer single chain, Si-methyl terminated of 507 atoms each (Figure 2).

To create a bulky mass, an NPT stage was carried by rising the pressure up to 1.5 GPa for 500 ps to set the density of the polymer to about 0.97 g cm^−3^, a typical literature value [32]. All the interatomic forces were calculated with the COMPASS force field from Sun and coworkers [33]. COMPASS is a Class-2 force field which provides a detailed representation of bond and non-bond interaction for soft matter and it has already been used for PDMS in thermal applications [34]. The COMPASS force field was also used for the whole system due to the primary bonding between graphene and molecular junctions, based on our previous work [19,22]. The msi2lmp tool included in LAMMPS package was used to produce the input data files with corresponding atomic interactions on the basis of COMPASS force field. The central part of the model was constituted by a couple of 95 × 25 Å^2^ graphene platelets jointed by three molecular junctions covalently bound, plus an unjointed one for comparison. Overall, about 36,500 atoms were computed in each simulation. As a common assumption, the van der Waals cut-off was set to 10 Å.

**Figure 3:**
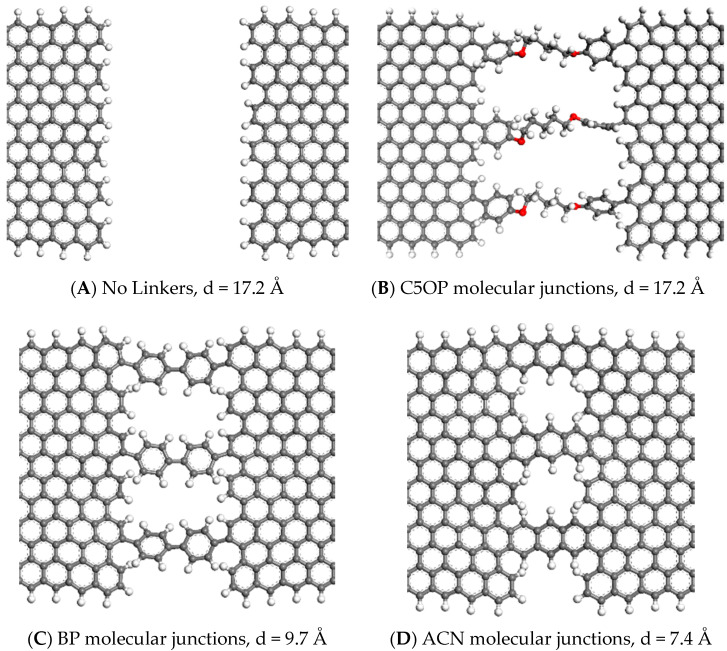
Detail of the molecular junctions connecting graphene sheets in polymer bound models. (**A**) is the junction without linkers (No Linkers) while three molecules constitute the junction made of (**B**) C5OP, (**C**) BP and (**D**) ACN. The letter “d” indicates the distance between the graphene edges. Color codes: carbon atoms in grey, oxygen in red, hydrogen in white.

Three different molecular structures were selected in this work as junctions between the graphene foils and compared with the unjointed graphene. We choose the three molecules based on the results from our previous studies where we screened some chemically feasible [22] thermal linkers without any polymer interaction [19]. In this work, the first case study is the unjointed model made of two graphene platelets—it was designed to represent the typical contact between graphene flakes and the surrounding polymer matrix, and it is referred as “No Linkers” (Figure 3A). The first molecule acting as a linker is 1,5-diphenoxypentane (C5OP), a hybrid molecule made by two segments, an aliphatic and aromatic one (Figure 3B). The second junction is biphenyl, (BP, Figure 3C), which belongs to the family of the aromatic molecules, and third, the anthracene (ACN, Figure 3D), is a polyaromatic molecule, representing an ideal junction, both in terms of conformational stiffness and similarity to graphene, yet challenging to prepare experimentally.

**Figure 4:**
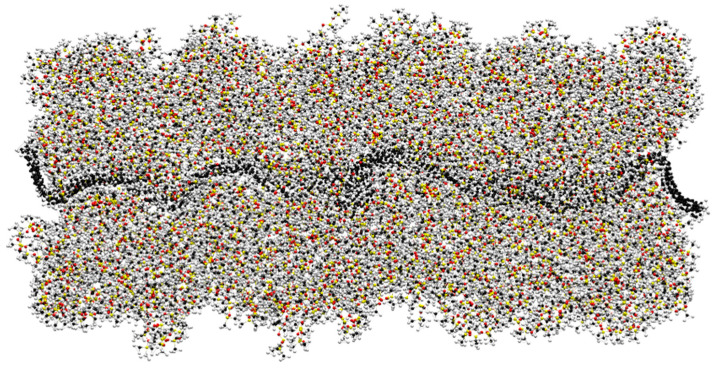
PDMS polymer surrounding graphene flakes jointed by three C5OP linkers hindered by the polymer). Color codes: carbon atoms in black, oxygen in red, hydrogen in light gray and silicon in yellow.

Figure 4 represents how the polymer embeds the graphene junction during the simulation. The topology appears different to the one reported previously in Figure 1. This is due to the strong interaction of the graphene sheets with the PDMS molecules [35]. The larger mass of PDMS, in fact, forces the thin graphene sheets (including the junction) to fold and crease. This fact is realistic and well representative of actual flexible graphene sheets dispersed in a polymer matrix [36], as already observed through transmission electron microscopy [37,38,39]. It is worth mentioning that molecular junctions are also bent as a consequence of graphene deformation. While the conformation of flexible molecular junctions was previously reported to affect their thermal conductance [20], the calculation of thermal conductance for PDMS embedded junctions is performed by averaging the contribution of the different junctions, having different conformations within the simulation cell, as well as averaging the three replicas.

## 4. Results and Discussion

**Figure 5:**
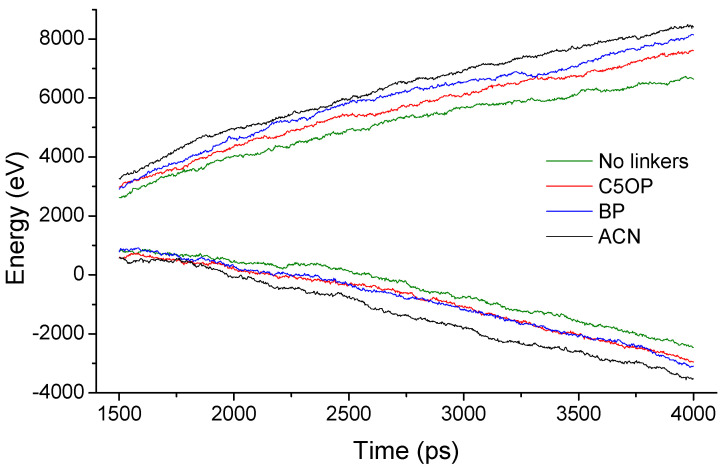
Energy flowing through thermostats as a function of the time for PDMS-surrounded graphene interfaces. Four simulations are taken as examples for three different linkers (C5OP, BP, and ACN) and a linkerless model (No linkers).

For each of the molecular junction described above, the energy transferred from the hot thermostat region to the cold thermostat region is reported in Figure 5. In this figure, the plots for the upper branch correspond to the injected heat from the heat source, while the lower branch represents the energy drained from the heat sink. Our results exhibit an almost linear trend with some fluctuations due to the small number of atoms belonging to each thermal slab (approx. 100) and limited slope differences, within ±10% from the average value, as summarized in Table 1. The energy flux slopes herein are obtained for three sets of replicas, each adopting a different seed for the initial velocities. Within the same set of data, the uncertainty from differences in slope between the upper and lower branches (thermostats variation from the average value) and the uncertainty from replication (replica error from the average value) are included. The last column of Table 1 indicates the enhancement from the linker adoption over the linkerless condition as the ratio between the computed heat flux enhancements from the linker adoption, over the linkerless condition. The amount of the transferred heat reported in Table 1 indicates that the presence of molecular linkers induces an overall improvement in thermal transport between the graphene flakes. In fact, the aliphatic/aromatic C5OP molecular junction determines a moderate increase in heat flux, rising from 1.47 to 1.53 eV ps^−1^ with an average improvement of about 4%. For the aromatic biphenyl, the heat flux rises up to 1.65 eV ps^−1^ and for the anthracene, a value of 1.78 eV ps^−1^ was calculated, with a gain of about 12% and 21%, respectively.

**Figure 6:**
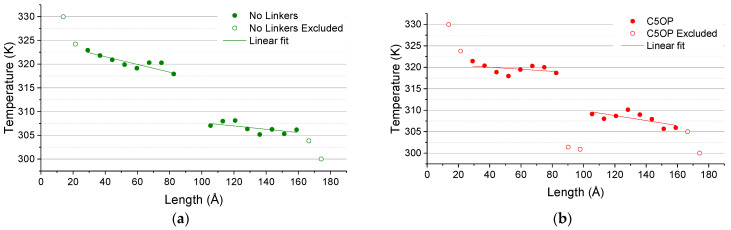

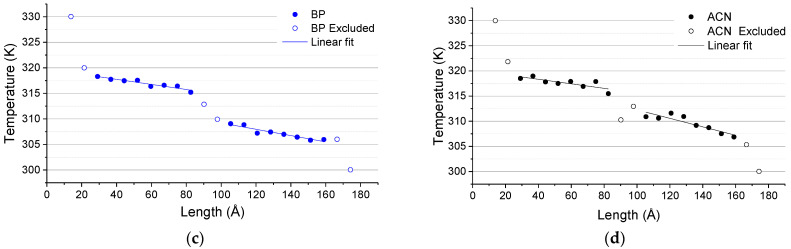
Temperature as a function of the slabs length without molecular junction (**a**), with C5OP (**b**), with biphenyl (**c**) anthracene (**d**) junctions, embedded in PDMS. Empty dots are values excluded from data fitting due to non-linearities.

The temperatures of the graphene thermal layers for PDMS-embedded junctions investigated are reported in Figure 6, where empty dots indicate the points not included in the fitting calculation, such as the one close to thermostats or across the junction.

**Figure 7:**
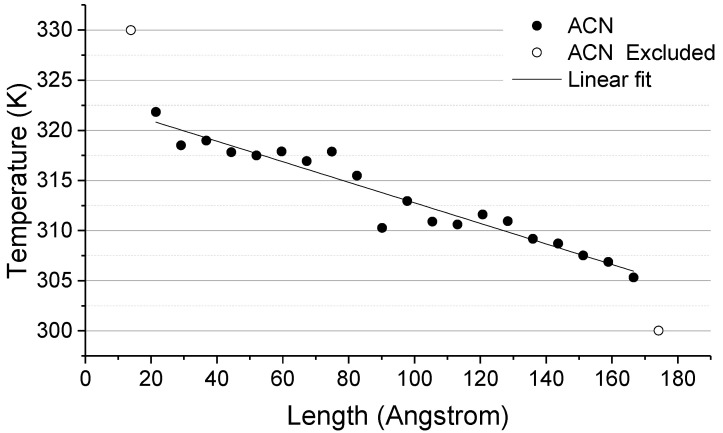
Temperature of thermal slabs as a function of the position. The linear fit among anthracene (ACN) slabs temperature suggests the suppression of the thermal jump across the junction.

The model without molecular junctions between the graphene foils (Figure 6a) reported an average temperature jump, as evaluated from the projection of the slopes of about 8.65 ± 0.57 K. By the heat flux calculation reported in Table 1 and the temperature difference across the junction (Table 2), the thermal conductance was found to be about 288 ± 21 MW m^−2^ K^−1^. As a general physical concept, with longer or more complex paths for the phonons to transport, the phonon scattering increases and thus the lower is expected to be the thermal conductance. Such a basic concept is in agreement with our findings as summarized in Table 2, in which the drop of thermal conductance from ACN to BP and C5OP molecular junctions is observable. Is noteworthy that all the conductance values reported herein are calculated by considering the whole interface between the graphene flakes and PDMS as the contact area (9500 Å^2^), thus representing the conductance of the whole system between the two thermostats as a contribution of ITC between graphene and PDMS and TBC trough edges. Adopting the same methodology for the other models where linkers were employed, the C5OP temperature jump (Figure 6b and Table 2) was calculated to be about 7.46 ± 0.31 K, and consequently the thermal conductance increased to 346 ± 26 MW m^−2^ K^−1^. By the use of C5OP linkers, the temperature across the interface became the 86% of the initial value when no linkers were adopted. Thus, we can assume that the reduction in thermal jump is responsible for the overall increase in thermal conductance, quantified in about 20% for C5OP. The temperature trend observed with the biphenyl junction (Figure 6c) shows a temperature jump reduced by approx. one third compared to the linkerless model, decreasing from 8.65 ± 0.57 K to 5.78 ± 0.40 K (Table 2). The temperature plot indicated a strong coupling between the graphene flakes, where the calculated temperature of the junction progressively matches the linear fit of the temperature slabs in graphene. Overall, for the three replicas, biphenyl jointed models reported a calculated thermal conductance of about 484 ± 55 MW m^−2^ K^−1^, 68% more than the linkerless model, mainly from the contribution of thermal jump reduction (67%, Table 2) than increased heat transferred, slightly over 12% (Table 1). The anthracene junction was also investigated with the purpose of finding the upper-bound condition, i.e., a sort of ideal molecular junction structure, regardless of its experimental viability. The temperature of the junction as a function of the displacement is reported in Figure 6dd. In this case, the thermal jump (2.03 ± 0.32 K) is significantly smaller than with the previous junctions, leading to a thermal conductance of about 4.7 times (1363 ± 18 MW m^−2^ K^−1^) the thermal conductance of the linkerless junction. Given the small thermal jump observed in the acene-bound interface, the ACN junction could be considered the equivalent of a continuous defective material. With this latter model, a continuous fit of the temperature may be applied, as depicted in Figure 7. From the application of Fourier’s law, the thermal conductivity of the joint graphene slabs was calculated at about 332 W m^−1^ K^−1^. This value may be considered representative of a network of graphene flakes fully joined by acene junctions within a PDMS matrix and represents a theoretical upper value for molecularly joined graphene-based nanocomposites.

While the thermal conductance calculated for the different PDMS embedded molecular junctions suggest large differences in their effectiveness, the conductance trend appears coherent with conductance values reported for self-standing junctions [19]. However, splitting the contribute in thermal transport from the PDMS chains and the molecular junctions is not trivial, because the calculated thermal conductance are dependent on the layout, in particular for the different heat transfer areas, a parameter that cannot be accurately quantified in molecules. According to previous results [19], the heat flux for three molecules of C5OP, BP and ACN, is about 0.05, 0.17 and 0.35 eV/ps, respectively. Table 3 reports the contribution from the matrix, which corresponds to the “No Linkers” model and the self-standing molecules evaluated in our previous work [19]. From this comparison, the thermal contribution of the linkers and matrix appears to be roughly cumulative, with values that agrees with the results proposed previously. This result indicates that the introduction of thermal linkers could be an effective strategy to reduce the thermal resistance at the interface inside polymeric materials. Moreover, their contribution appears unaffected by the deformation of the structure during the simulation, a condition that typically occurs in actual nanocomposites.

## 5. Conclusions

The thermal conductance between graphene platelets surrounded by a PDMS polymer matrix has been evaluated trough NEMD simulations. We observed that even in presence of a tight contact with the polymer, the linker type influenced the overall conductance value by reducing the thermal drop. The hybrid aliphatic and aromatic C5OP junction yields to a thermal conductance improvement of about 20% compared to the unbound sheets, while aromatic structures provided higher enhancements, confirming the previously reported trends for self-standing junctions. In particular, biphenyl junctions enhanced thermal conductance by about 68% while anthracene linkers, considered as a sort of upper bound theoretical case study, yielded to a five-times reduction of thermal resistance. We also observed that the presence of the molecular junctions contributes to the total heat flux, adding on the heat flow exchanged via the surrounding polymer, embedding the junctions. The strong dependence of the thermal conductance in PDMS and graphene model is attributed to both the length and the chemical structure of the linker. On one hand, short linkers force the flakes to keep a smaller gap between the platelets than longer ones; consequently, the volume of interposed polymer is smaller. On the other hand, the length of the linker is not sufficient to justify the observations and it is therefore suggested that the conformational stiffness of the linker strongly affects the efficiency of the junction, in agreement with previous findings for self-standing junctions. The findings reported in this paper are therefore opening the way for the engineering of thermal contacts between conductive nanoparticles, within the next generation of advanced polymer based materials for heat management applications.

## Figures and Tables

**Table 1:** Averaged heat flux for different linkers in PDMS. Average value, thermostat variation, replica variation and linker gain as the ratio of linker heat flux over linkerless heat flux value.

Linker Type	Average Value	Thermostat Upper/Lower Branch Variation	Replica Variation	Linker Gain
	[eV/ps]	[eV/ps]	[eV/ps]	[%]
No linkers	1.47	±0.07	±0.02	−
C5OP	1.53	±0.12	±0.09	+4
BP	1.65	±0.14	±0.08	+12
ACN	1.78	±0.11	±0.06	+21

**Table 2:** Thermal jump, thermal jump reduction, thermal conductance and thermal conductance change, for all the systems proposed.

Linker Type	Average Thermal Jump ± Replica Variation [K]	Ratio to “No Linkers”	Thermal Conductance [MW m^−2^ K^−1^]	Ratio to “No Linkers”
No linkers	8.65 ± 0.57	1.00	288 ± 21	1.00
C5OP	7.46 ± 0.31	0.86	346 ± 26	1.20
BP	5.78 ± 0.40	0.67	484 ± 55	1.68
ACN	2.03 ± 0.32	0.23	1363 ± 18	4.73

**Table 3:** Heat flux from the cumulative calculations (as reported in Table 1) and separate contribution from linkers and matrix.

Junction Type	Energy Flux [eV/ps]		
	Cumulative Calculation (This Work)	Self-Standing Linkers Contribution (from [19])	No Linkers (This Work) + Self-Standing Linkers Contribution
No linkers	1.47 ± 0.07	-	-
C5OP	1.53 ± 0.12	0.05	1.47 + 0.05 = 1.52 (−0.6%)
BP	1.65 ± 0.14	0.17	1.47 + 0.17 = 1.64 (−0.6%)
ACN	1.78 ± 0.11	0.35	1.47 + 0.35 = 1.82 (+2.2%)

## Data Availability

The data is available on reasonable request from the authors.

## Abbreviations

PDMS	Polydimethylsiloxane
MD	Molecular Dynamics
NEMD	Non-Equilibrium Molecular Dynamics
NVT	Canonical statistical ensemble (constant Number of particles, Volume and Temperature)
NVE	<+Microcanonical statistical ensemble (constant Number of particles, Volume and Energy)
NPT	Isothermal–isobaric statistical ensemble (constant Number of atoms, Pressure and Temperature)
C5OP	1,5-diphenoxypentane
BP	Biphenyl
ACN	Anthracene
